# In-situ TEM observation of stacking-fault intersection–controlled partial dislocation dynamics in high-Mn austenitic steel

**DOI:** 10.1186/s42649-026-00135-9

**Published:** 2026-05-07

**Authors:** Sung-Dae Kim

**Affiliations:** Department of Materials Science and Engineering, Pukyong National University, Busan, 48513 Republic of Korea

**Keywords:** High-manganese steel, In-situ TEM, Stacking fault interaction, Partial dislocation, Dislocation interaction

## Abstract

The deformation behavior of a high-Mn austenitic steel was investigated using in-situ straining transmission electron microscopy (TEM), with emphasis on the dynamic interaction between stacking faults and partial dislocations during plastic deformation. Owing to the low stacking-fault energy of the alloy, plastic deformation is primarily governed by Shockley partial dislocations, leading to extensive stacking-fault formation on multiple {111} planes. As deformation proceeds, stacking faults generated on different slip variants frequently intersect within grain interiors. Real-time observations reveal that these intersections act as strong deformation-induced barriers that impede partial dislocation motion, resulting in dislocation pile-up and localized strain concentration. Such interactions are considered to contribute significantly to strain hardening in low stacking-fault energy austenitic alloys. Despite their strong blocking effect, stacking-fault intersections are not strictly impenetrable. Under conditions of significant dislocation accumulation, the separation distance between leading and trailing partial dislocations can locally decrease, allowing temporary recombination into a perfect dislocation segment. The recombined dislocation adopts screw character, enabling cross-slip onto a secondary {111} slip plane, followed by re-dissociation into Shockley partial dislocations.These findings demonstrate that stacking-fault intersections function as dynamic microstructural features that both impede and mediate dislocation motion. The present in-situ TEM observations provide direct mechanistic insight into stacking-fault intersection-controlled dislocation dynamics and their role in strain hardening and local plastic accommodation in low stacking-fault energy austenitic steels.

**Supplementary Information:**

The online version contains supplementary material available at 10.1186/s42649-026-00135-9.

## Introduction

High-manganese austenitic steels are widely studied for their exceptional combination of strength and ductility, which originates from their ability to maintain a high strain-hardening rate during plastic deformation (Grässel et al. 2000). In low stacking-fault energy austenitic steels, this behavior is closely related to the stacking fault energy (SFE) of austenite, which governs partial dislocation activity, stacking fault formation, and strain-induced ε-martensitic transformation (Park et al. 2010; Pierce et al. 2014; Gutierrez-Urrutia and Raabe 2012; Byun 2003).

In low-SFE austenitic steels, plastic deformation is primarily accommodated by Shockley partial dislocations, leading to the accumulation of stacking faults on {111} planes. With increasing strain, interactions among stacking faults can promote the formation of continuous fault sequences that serve as precursors for ε-martensite (Allain et al. 2004; Kim et al. 2020; Cooman et al. 2018; Ko et al. 2022). While the role of stacking faults in deformation behavior is well recognized, the dynamic interactions between stacking faults formed on different {111} planes and their influence on partial dislocation mobility remain insufficiently understood.

Most previous investigations have relied on post-mortem microstructural characterization, which provides only static information after deformation (Idrissi et al. 2010; Koyama et al. 2011; Talonen and Hänninen 2007). Consequently, direct experimental evidence linking stacking-fault interactions to dislocation-level strain hardening is limited. In particular, the role of stacking-fault intersections as dynamic obstacles to partial dislocation motion, and the conditions under which partial dislocations may locally overcome these obstacles, have not been clearly established.

In this study, in-situ straining transmission electron microscopy is employed to directly observe the deformation behavior of a high-Mn steel in real time. The evolution of partial dislocations, stacking faults, and their intersections on multiple {111} planes is captured during deformation. The results demonstrate that stacking-fault intersections act as dominant, deformation-generated barriers to partial dislocation motion, thereby enhancing work hardening, while also permitting localized and transient cross-slip under extreme local stress conditions. These findings provide new mechanistic insight into the interplay between stacking-fault interactions, strain hardening, and transformation behavior in high-Mn steels.

## Experimental methods

A representative high-manganese austenitic steel was selected for the present study. The nominal chemical composition was Fe–25Mn–0.4C (wt.%), designed to stabilize austenite at room temperature while maintaining a low stacking fault energy (SFE) favorable for stacking fault formation and strain-induced ε-martensitic transformation. The alloy was produced by vacuum induction melting and cast into ingots. After homogenization at 1200 °C for 5 h, the ingots were hot-forged into plates and solution-treated at 1050 °C for 1 h followed by water quenching to suppress carbide precipitation and retain a fully austenitic microstructure prior to deformation.

Thin foils for transmission electron microscopy were prepared by mechanical grinding followed by twin-jet electrochemical polishing. For in-situ straining TEM experiments, elongated strip-type specimens (~ 3 mm × 12 mm) were fabricated from the polished foils using a custom punching method. The specimen geometry and preparation procedure were adapted from previously reported in-situ TEM studies on high-Mn steels. The punched samples were electropolished using a perchloric acid–methanol (9:1) solution at − 20 °C to obtain electron-transparent regions (TenuPol-5, Struers). For post-mortem analysis of the deformed samples, discs with a diameter of 3 mm were punched from the deformed samples.

Transmission electron microscopy was carried out at an accelerating voltage of 200 kV (JEM-2100F, JEOL Ltd.). Bright-field (BF) imaging conditions were primarily employed to visualize dislocation activity and stacking fault formation with high temporal resolution. In-situ straining TEM experiments were performed using a dedicated straining holder (model 654, Gatan), enabling controlled tensile deformation inside the TEM column. Deformation was applied under displacement-controlled conditions to allow quasi-static loading while continuously observing microstructural evolution. Real-time TEM videos were recorded during deformation to capture the nucleation, glide, and interaction of partial dislocations and the associated formation of stacking faults (Camtasia studio, TechSmith). Observations focused on grains oriented near low-index zone axes, enabling clear identification of stacking faults formed on different {111} planes and their intersections. Low-angle annular dark-field scanning transmission electron microscopy (LAADF-STEM) images were acquired using an annular detector with a typical inner collection angle of ~ 20–30 mrad, providing contrast sensitive to lattice strain and atomic displacement. Regions containing intersecting stacking faults were identified by conventional TEM and subsequently examined by LAADF-STEM.

To clarify the deformation conditions during the in-situ TEM experiments, the applied strain rate was estimated to be on the order of ~ 10⁻^4^–10⁻^3^ s⁻^1^ based on the displacement rate and specimen geometry, which is within the typical range used in in-situ TEM deformation experiments. The initial microstructure of the investigated alloy was carefully examined prior to deformation and confirmed to consist predominantly of FCC austenite, with no detectable presence of thermally induced martensite within the resolution of the present analysis. These conditions provide a well-defined starting point for interpreting the observed stacking-fault formation and dislocation interactions during deformation.

## Results and discussion

**Fig. 1:**
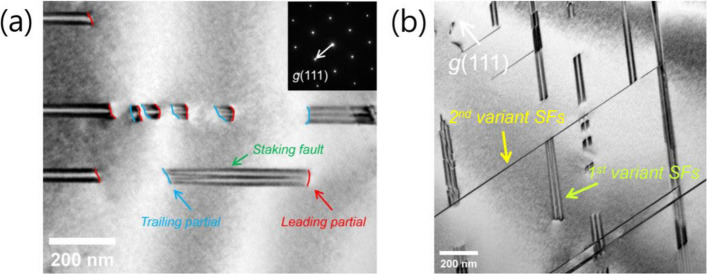
**a** Bright-field TEM image showing the nucleation and glide of Shockley partial dislocations after yielding. Each partial leaves behind a stacking fault (SF), indicating that plastic deformation is primarily accommodated through stacking-fault formation. **b** Formation of stacking faults on multiple {111} planes within a single grain. Activation of different slip variants results in intersecting stacking faults, establishing a three-dimensional fault network during deformation

Figure 1 and Supplementary Video S1 demonstrate that plastic deformation in the investigated high-Mn steel is governed predominantly by Shockley partial dislocation activity. Immediately after yielding, numerous dislocation segments are nucleated and propagate over extended distances (Fig. 1a). Burgers vector analysis confirms that these dislocations correspond to 1/6⟨112⟩ Shockley partials rather than perfect dislocations. Each moving partial leaves behind a planar defect consistent with a stacking fault (SF), indicating that plastic strain is primarily accommodated through stacking-fault formation. The dominance of partial dislocations is consistent with the low stacking-fault energy (SFE) of the present alloy (~ 20 mJ·m⁻^2^), which promotes wide dissociation of perfect dislocations into leading and trailing partials (Hull and Bacon 2011; Dumay et al. 2008). In contrast to moderate- or high-SFE FCC alloys—where the trailing partial readily follows the leading partial and restores perfect stacking—the present in-situ observations show that trailing partial motion is frequently suppressed. Consequently, stacking faults remain stable and accumulate progressively with increasing strain. As deformation proceeds, SFs form on multiple {111} planes within individual grains (Fig. 1b). Activation of multiple slip variants leads to the development of a three-dimensional network of intersecting stacking faults. This multi-variant stacking-fault configuration provides the microstructural framework for the intersection-controlled deformation behavior discussed below.

The average grain size of the investigated alloy was measured to be ~ 15 μm. In low stacking-fault energy austenitic alloys, grain boundaries are known to serve as important sources for the emission of Shockley partial dislocations and play a significant role in the formation of stacking faults. In the present study, the observed stacking-fault formation and subsequent dislocation interactions are therefore considered to be influenced, at least in part, by grain-boundary-mediated dislocation nucleation. Furthermore, the grain size may affect the frequency of dislocation–SF interactions by controlling the mean free path of dislocations, thereby contributing to the development of localized deformation structures and strain hardening behavior.

**Fig. 2:**
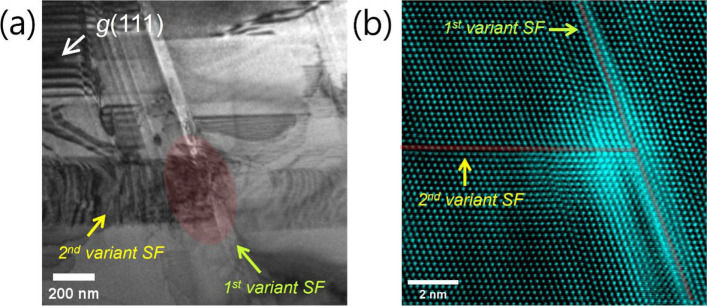
**a** In-situ TEM image showing a partial dislocation gliding on a {111} plane being arrested at the geometric intersection with a stacking fault formed on a different {111} variant (highlighted region). Real-time observation confirms that the obstruction occurs precisely at the intersection site. **b** LAADF-STEM image of an SF intersection. The enhanced intensity at the intersection indicates significant local lattice distortion and strain accumulation, supporting its role as a strong deformation-generated barrier to partial dislocation motion

During continued straining, partial dislocations gliding on a given {111} plane are frequently observed to decelerate or become arrested when encountering a stacking fault formed on a different {111} plane (Fig. 2a). Because the process is captured in real time (Supplementary Video S2), the causal relationship is unequivocal: obstruction occurs precisely at the geometric intersection between stacking faults. These interactions take place entirely within grain interiors and evolve dynamically during deformation, ruling out conventional obstacles such as precipitates or grain boundaries. This blocking behavior is consistent with the established understanding that Shockley partial dislocations are crystallographically confined to their original {111} planes and possess intrinsically limited cross-slip capability in low-SFE alloys. Cross-slip requires recombination of leading and trailing partials into a perfect dislocation, followed by redissociation on another {111} plane—a process that is energetically unfavorable under typical conditions (Hull and Bacon 2011; Hirth and Lothe 1982). Consequently, stacking-fault intersections act as strong geometric barriers to partial dislocation glide. LAADF-STEM analysis further supports this interpretation. In LAADF mode, image intensity is sensitive to strain-induced diffuse scattering; regions exhibiting greater lattice distortion appear brighter (Pennycook and Nellist 2011; Batson et al. 2002). The pronounced intensity enhancement observed at SF intersections (Fig. 2b) indicates significant local strain accumulation. Such strain concentration further increases the energetic barrier for dislocation transmission across the intersection. As deformation proceeds and the density of stacking faults increases, the number of intersection sites correspondingly rises. These intersections therefore constitute deformation-generated obstacles that continuously emerge during plastic flow. The resulting accumulation of partial dislocations at these sites enhances local stress concentration and contributes substantially to the high work-hardening capability characteristic of low-SFE austenitic steels. Up to this point, the present observations directly confirm the widely recognized role of stacking-fault intersections as strengthening elements in low-SFE alloys (Grässel et al. 2000; Park et al. 2010; Pierce et al. 2014; Gutierrez-Urrutia and Raabe 2012; Allain et al. 2004; Kim et al. 2020; Cooman et al. 2018; Ko et al. 2022; Kim et al. 2018; Li et al. 2020).

**Fig. 3:**
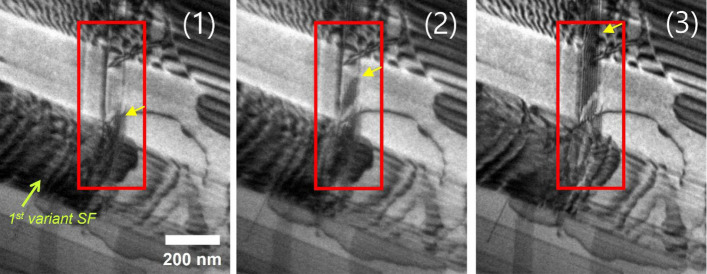
Sequential TEM images showing (i) accumulation of partial dislocations at the intersection, (ii) stress-assisted recombination of leading and trailing partials into a perfect dislocation segment, (iii) formation of a screw-type perfect dislocation (u ∥ b), and (iv) cross-slip onto a secondary {111} plane. After cross-slip, the dislocation redissociates into partial dislocations on the new slip plane

**Fig. 4:**
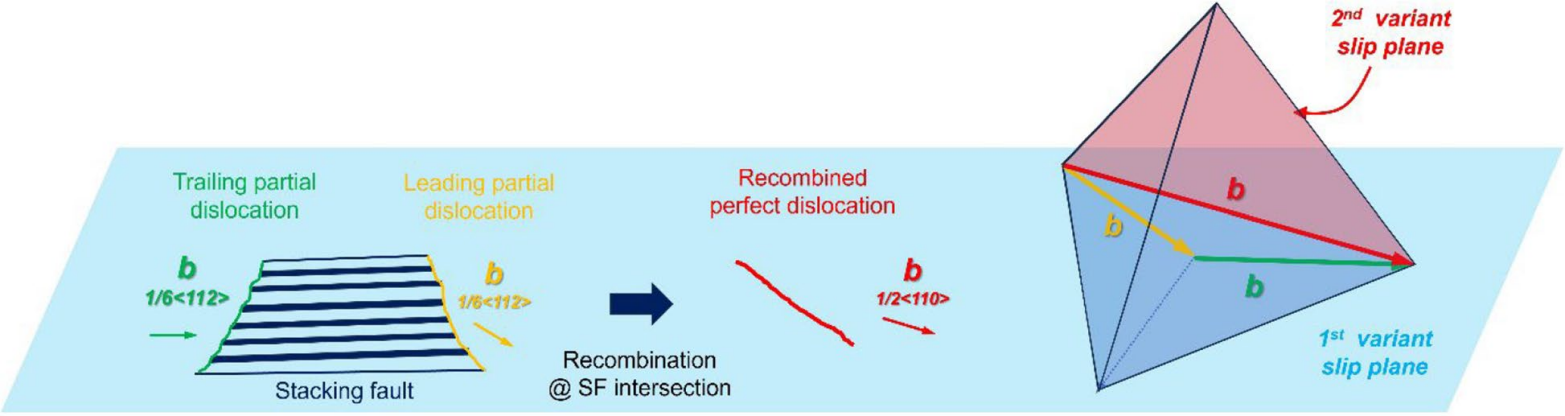
Schematic illustration of dislocation evolution at a stacking-fault intersection represented on a Thomson tetrahedron. Partial dislocations gliding on a primary {111} plane accumulate at the intersection and recombine to form a perfect dislocation with a ⟨110⟩ Burgers vector. Owing to the intersection geometry, the recombined segment assumes screw character (u∥ b), enabling cross-slip onto a secondary {111} plane. On the secondary plane, the perfect dislocation redissociates into leading and trailing Shockley partials due to the low stacking-fault energy of the alloy

While stacking-fault intersections primarily function as strong barriers, detailed in-situ observations (Supplementary Videos S3 & S4) reveal that they are not strictly impenetrable. In a limited number of cases, localized and transient cross-slip events are observed at intersection sites after significant dislocation accumulation. Frame-by-frame analysis (Fig. 3) indicates that such events occur only after pronounced pile-up of partial dislocations at a given intersection. The resulting stress concentration locally reduces the separation distance between the leading and trailing partials, facilitating their temporary recombination into a perfect dislocation segment. Importantly, this recombined segment remains pinned at the intersection, which serves as a fixed anchoring point. According to the Thomson tetrahedron construction, recombination of two Shockley partial dislocations on a given {111} slip system produces a perfect dislocation with a ⟨110⟩ Burgers vector (b). At the intersection geometry observed here, the line direction (u) of the recombined segment coincides with this ⟨110⟩ direction. Consequently, the recombined perfect dislocation inherently assumes screw character, with the line vector (u) parallel to the Burgers vector (b) (i.e., u ∥ b) immediately upon recombination. Because screw dislocations in FCC crystals can cross-slip between equivalent {111} planes, this configuration renders cross-slip onto a secondary {111} variant crystallographically permissible (Hull and Bacon 2011; Hirth and Lothe 1982). Once transferred to the secondary slip plane, the perfect dislocation segment can again dissociate into leading and trailing Shockley partials due to the low stacking-fault energy of the alloy. The re-formed partial dislocations then resume glide on the secondary variant, leaving behind a stacking fault on that plane. Thus, the observed cross-slip originates from partial recombination that inherently produces a screw-type perfect dislocation at the intersection. The overall sequence can therefore be summarized as (Fig. 4): partial blocking → stress accumulation → recombination into a screw-type perfect dislocation → cross-slip → redissociation into partial dislocations on the secondary plane.

The observed deformation behavior can be interpreted in terms of the evolution of deformation mechanisms with increasing strain. In the early stage of deformation, plasticity is primarily governed by the glide of dislocations, with relatively limited stacking-fault formation. As deformation proceeds, the formation and interaction of stacking faults become more pronounced, leading to frequent encounters between gliding partial dislocations and pre-existing SFs. In this regime, stacking-fault intersections play an increasingly important role by impeding dislocation motion, promoting dislocation accumulation, and generating localized stress concentration. These effects contribute to enhanced strain hardening. Although direct macroscopic stress–strain data are not available from the present in-situ TEM experiments, the observed sequence of dislocation activities is consistent with deformation behavior typically reported for low stacking-fault energy austenitic alloys.

The present results refine the conventional picture of stacking-fault intersections in low-SFE austenitic steels. Their primary and dominant role is to impede partial dislocation motion, promote dislocation pile-up, and enhance work hardening. This strengthening effect arises from both crystallographic confinement of partial dislocations and strain concentration at intersection sites. However, the in-situ observations demonstrate that under sufficiently high local stress, these same intersections can act as sites of stress-induced dislocation character transformation. The inability of partial dislocations to readily cross-slip enables substantial stress accumulation, which in turn drives recombination and screw-segment formation, ultimately permitting localized cross-slip. Therefore, stacking-fault intersections exhibit a dual but asymmetric mechanical function: they primarily serve as strengthening barriers, while secondarily enabling stress-activated dislocation reconfiguration when a critical local stress threshold is exceeded. The cross-slip events are spatially limited and transient, and do not negate the overall hardening effect; instead, they provide a localized stress-relief pathway within an otherwise strongly hardened microstructure. By directly visualizing this dynamic transition in real time, the present study extends the understanding of intersection-controlled plasticity beyond static blocking and reveals the underlying mechanism by which extreme local stresses can trigger dislocation character transformation in low-SFE austenitic steels.

It should be noted that the present observations were obtained from thin TEM specimens, where free surface effects and reduced constraint may influence dislocation behavior. In particular, image forces and surface relaxation can modify the local stress state, potentially facilitating dislocation escape and altering the apparent strength of obstacles such as stacking-fault intersections. Furthermore, cross-slip behavior may be affected by the reduced dimensional constraint in thin foils. Therefore, caution is required when directly extrapolating these observations to bulk deformation behavior. Nevertheless, the fundamental interaction mechanisms identified in this study are expected to remain qualitatively relevant.

The influence of interstitial carbon on stacking-fault formation and dislocation behavior should also be considered. Carbon is known to affect stacking-fault stability, dislocation mobility, and local stress fields in austenitic steels. Although these effects may influence the formation and interaction of stacking faults, including their intersection behavior, the present study does not provide a direct assessment of carbon-related effects. Therefore, a systematic investigation of the role of carbon concentration in stacking-fault formation and evolution is required in future work.

## Conclusions

In this study, the deformation behavior of a high-Mn steel was investigated by in-situ straining transmission electron microscopy, with particular focus on the dynamic role of stacking-fault (SF) intersections during plastic deformation. The main findings are summarized as follows:

1. Plastic deformation is dominated by Shockley partial dislocations due to the low stacking-fault energy of the alloy, leading to extensive stacking-fault formation on multiple {111} planes and the development of a three-dimensional network of intersecting faults.
2. Stacking faults formed on different {111} planes frequently intersect within grain interiors. Real-time TEM observations directly confirm that these intersections act as strong deformation-generated barriers to partial dislocation motion. The resulting dislocation pile-up and local strain concentration contribute significantly to the pronounced work-hardening behavior of the high-Mn steel steel.
3. Although SF intersections primarily function as strengthening obstacles, localized and transient cross-slip events can occur under sufficiently high local stress conditions. These events are initiated by stress-assisted recombination of leading and trailing partial dislocations into a perfect dislocation segment, followed by rotation of the dislocation line into screw character (u ∥ b), which enables cross-slip onto an alternative {111} plane.
4. The observed cross-slip does not reflect intrinsically facile partial dislocation motion in low-SFE alloys. Rather, it represents a stress-activated dislocation character transformation that occurs only after substantial stress accumulation at SF intersections.

Overall, the present in-situ observations demonstrate that stacking-fault intersections in high-Mn steel exhibit a dual but asymmetric mechanical role: they primarily enhance strength by blocking partial dislocation glide, while secondarily providing a localized, stress-driven pathway for dislocation reconfiguration. These findings refine the mechanistic understanding of intersection-controlled plasticity and highlight the dynamic nature of dislocation processes in low-SFE austenitic steels.

## Supplementary Information

Supplementary Material 1: Supplementary Video S1: Real-time in-situ TEM observation of partial dislocation nucleation and glide in high-manganese steel during tensile deformation. The video shows the initial formation of stacking faults and the propagation of leading partial dislocations along {111} slip planes.

Supplementary Material 2: Supplementary Video S2: In-situ TEM recording showing interactions between moving partial dislocations and pre-existing stacking faults. Dislocation blocking and pile-up at stacking-fault intersections are observed, illustrating the role of stacking-fault interactions in strain hardening.

Supplementary Material 3: Supplementary Video S3: In-situ TEM observation of the same mechanism, highlighting the cross-slip process of partial dislocations initially pinned at the stacking-fault intersection. After cross slip to another variant, the dislocations propagate and generate a stacking fault on the adjacent plane.

Supplementary Material 4: Supplementary Video S4: In-situ TEM observation of the same mechanism, highlighting the cross-slip process of partial dislocations initially pinned at the stacking-fault intersection. After cross slip to another variant, the dislocations propagate and generate a stacking fault on the adjacent plane.

## Data Availability

The datasets generated during the current study are available from the corresponding author on reasonable request.
